# Postcoital Bioavailability and Antiviral Activity of 0.5% PRO 2000 Gel: Implications for Future Microbicide Clinical Trials

**DOI:** 10.1371/journal.pone.0008781

**Published:** 2010-01-22

**Authors:** Marla J. Keller, Pedro M. M. Mesquita, N. Merna Torres, Sylvia Cho, Gail Shust, Rebecca P. Madan, Hillel W. Cohen, Julie Petrie, Tara Ford, Lydia Soto-Torres, Albert T. Profy, Betsy C. Herold

**Affiliations:** 1 Department of Medicine, Albert Einstein College of Medicine, Bronx, New York, United States of America; 2 Department of Obstetrics and Gynecology and Women's Health, Albert Einstein College of Medicine, Bronx, New York, United States of America; 3 Department of Pediatrics, Albert Einstein College of Medicine, Bronx, New York, United States of America; 4 Department of Epidemiology and Population Health, Albert Einstein College of Medicine, Bronx, New York, United States of America; 5 Department of and Microbiology and Immunology, Albert Einstein College of Medicine, Bronx, New York, United States of America; 6 Division of AIDS, National Institute of Allergy and Infectious Disease, National Institutes of Health, Bethesda, Maryland, United States of America; 7 Endo Pharmaceuticals, Chadds Ford, Pennsylvania, United States of America; National Institutes of Health, United States of America

## Abstract

**Background:**

The pharmacokinetics and pharmacodynamics of vaginal microbicides are typically assessed among sexually abstinent women. However, the physical act of sex may modulate gel distribution, and preclinical studies demonstrate seminal plasma interferes with the antiviral activity of several microbicides. This study compared the biological activity and concentration of PRO 2000 in cervicovaginal lavage (CVL) collected in the absence or following coitus.

**Methods:**

CVL samples were collected from ten heterosexual couples at baseline, after sex, after a single dose of 0.5% PRO 2000 gel and sex, and after gel application without sex. The impact of CVL on HIV-1 infection of TZM-bl cells and HSV-2 infection of CaSki cells was monitored by luciferase and plaque assay, respectively. PRO 2000 concentrations were measured by fluorescence.

**Results:**

CVL collected after PRO 2000 application significantly inhibited HIV-1 and HSV-2 (p = 0.01). However, the antiviral activity was reduced following sex and no significant protective effect was observed in postcoital CVL obtained in the presence compared to the absence of PRO 2000 for HIV (p = 0.45) or HSV-2 (p = 0.56). Less PRO 2000 was recovered in postcoital CVL, which, in conjunction with interference by seminal plasma, may have contributed to lower antiviral activity.

**Conclusions:**

Postcoital responses to PRO 2000 differ from precoital measures and the results obtained may provide insights into the clinical trial findings in which there was no significant protection against HIV-1 or HSV-2. Postcoital studies should be incorporated into clinical studies before embarking on large-scale efficacy trials.

## Introduction

HIV prevention is a critical health priority. While vaccines hold promise, approaches to elicit protective immune responses remain elusive. Pre-exposure prophylaxis with orally administered antiretroviral drugs may prove effective, but there are substantial concerns for toxicities associated with long-term exposure and the risk for selecting resistant viral variants. Topically delivered drugs, designated microbicides, offer distinct advantages because they would limit the toxicities associated with systemic prophylaxis. However, significant protection has yet to be achieved by this approach. In two recently completed clinical trials, HIV Prevention Trial Network (HPTN) 035, which was conducted among 3087 women and Microbicides Development Program (MDP) 301, which was conducted among 9385 women, no protection in HIV or HSV-2 acquisition was observed among women randomized to coitally-dependent intravaginal application of PRO 2000 compared to placebo gel. While the HPTN 035 trial showed a 30% (p = 0.10) reduction in HIV in the 0.5% PRO 2000 compared to placebo gel arm, [1], the MDP trial results found no evidence that PRO 2000 gel is not effective at preventing HIV infection in women [2]. Specifically, in the primary efficacy analysis, the rate of new HIV infection was 4.5 per 100 person-years in the 0.5% PRO 2000 arm compared to 4.3 in the placebo arm. Notably, the MDP trial initially also included a 2% PRO 2000 gel arm, which was prematurely discontinued by an independent data safety monitoring committee in February, 2008 because it would have little chance to show effectiveness; the HIV infection rate in the 2% PRO 2000 arm was 4.7 per 100 person-years compared with 3.9 in placebo arm at that time, though the difference was not statistically significant (p = 0.239). Moreover, none of the previous microbicide clinical trials with other candidate drugs has demonstrated significant protection against HIV and in some trials, higher rates of infection were observed in the treatment compared to the placebo arm [3], [4], [5]. Reduced potency against R5 viruses and difficulties with adherence to coitally-dependent products may have contributed to the lack of efficacy observed with some products. In addition, *in vitro* studies have shown that low concentrations of polyanions can increase HIV infection [6]. Furthermore, the induction of inflammatory responses and interference with innate mucosal defenses may have contributed to the enhanced HIV acquisition rates observed with nonoxynol-9 (N-9) and cellulose sulfate [7], [8].

Additional factors that may impact microbicide efficacy are sexual activity and semen. Preclinical studies of microbicides are typically performed in culture systems or animal models with virus introduced in buffer. These models do not reflect what occurs during heterosexual transmission, where female genital tract secretions, semen and coitus may modulate microbicide activity. Some microbicides show reduced antiviral effects in culture when virus is introduced in seminal plasma (SP) [9], [10]. For example, cervicovaginal lavage (CVL) obtained 1 hour post-application of 0.5% PRO 2000 gel inhibited HSV-2 infection ∼1000-fold when cells were infected with virus diluted in buffer, but by only 20-fold when virus was diluted in pooled human SP [10]. Mechanistic studies demonstrated that SP proteins competitively inhibited the binding of PRO 2000 to the HSV-2 envelope [10]. SP also interferes with the *in vitro* anti-HIV activity of some microbicides, although the mechanism(s) have not yet been elucidated and the extent of interference may be less than that observed with HSV-2 [9], [11]. Not only may semen interfere with antiviral activity, but semen-derived amyloid fibrils may enhance HIV infectivity, although the clinical relevance of these *in vitro* observations have not been substantiated [12].

Together, these observations suggest that the pharmacokinetics (PK) and pharmacodynamics (PD) of microbicides may differ following coitus. Thus, we performed an open-label study among 10 monogamous couples to determine the concentration of drug and antiviral activity in CVL obtained after intravaginal application of a single dose of 0.5% PRO 2000 gel in the absence of or following sexual intercourse. This study was completed prior to the release of the MDP301 results.

## Methods

### Ethics Statement

The study was approved by the Albert Einstein College of Medicine Institutional Review Board (IRB) and the NIAID Division of AIDS Prevention Science Review Committee; all participants provided written informed consent.

### Participants

Ten healthy couples were recruited between July 2008 and June 2009. Inclusion criteria included use of any form of contraception except condoms or a diaphragm and couples had to be at low risk for sexually transmitted infections (STI) (no STI in previous six months, no history of intravenous drug use, and in a mutually monogamous relationship for at least six months). Participants were excluded for pregnancy, breastfeeding, menopause, vasectomy, HIV, genitourinary infection, vaginitis or abnormal Pap test. Couples were also excluded if they were discordant with respect to HSV-1 or HSV-2 serostatus.

At screening, female participants had urine collected for microscopy and culture and blood was obtained for pregnancy testing. A gynecological examination was performed for detection of bacterial vaginosis (BV), *Trichomonas vaginalis*, and *Candida* species and a Pap test was collected. CVL was performed by washing the cervix and posterior fornix with 10 ml of normal saline (pH ∼5.5). Male subjects had a genital exam and urinalysis. All subjects had nucleic acid amplification testing of urine for *Neissseria gonorrhoeae* and *Chlamydia trachomatis* (Gen-Probe, Inc., San Diego, CA) and blood was collected for HIV ELISA, HIV-1 RNA by bDNA (Siemens Healthcare Diagnostics, Deerfield, IL), syphilis (rapid plasma reagin test), and serotype specific antibodies for HSV-1 and HSV-2 (HerpeSelect, Focus Diagnostics, Cypress, CA).

Subsequent study visits were conducted 3–5 days apart and participants were instructed to abstain from sex and ejaculation for 48 hours prior to each visit. At the second visit, couples were instructed to have vaginal-penile intercourse without using a condom either in a private room in the Clinical Research Center (CRC) or at home, if they lived in close proximity. Women were asked to return to the CRC for collection of a postcoital CVL within 2 hours. At the third visit, the study clinician applied a single dose of 0.5% PRO 2000 gel intravaginally, each couple was again instructed to have sex without using a condom and women were asked to return within 2 hours for a lavage. Only the females returned for the final visit at which a single dose of 0.5% PRO 2000 gel was applied intravaginally. The subject was then permitted to ambulate and return for collection of CVL at a time consistent with lavage collection at Visit 3.

Vaginal pH was measured from a swab of the lateral vaginal wall before and after coitus at Visit 2, before PRO 2000 gel application and after coitus at Visit 3 and before and after gel application at Visit 4 (Whatman pH paper, pH 3.8–5.5 and pHydrion paper, pH 6.0–9.5) (Couples 4–10). A swab of the vaginal wall was obtained following intercourse at Visits 2 and 3 to test for the presence of semen using an antibody immunoassay that detects p30, a glycoprotein produced by the prostate (Abacus Diagnostics, West Hills, CA).

### Study Drug

PRO 2000 gel is an aqueous gel containing 0.5% (w/w) PRO 2000 packaged in single dose (2-mL) lacquer-lined aluminum tubes and provided by Endo Pharmaceuticals, Chadds Ford, PA. Unformulated drug was also obtained from Endo.

### CVL and SP Samples

CVL were transported to the laboratory on ice and clarified by centrifugation at 700 rpm for 10 minutes at 4°C. Supernatants were divided into aliquots and stored at −80°C. The protein concentration (Pierce BCA) and pH (ColorpHast, pH 2–9, EMD Chemicals) were determined.

### HIV-Infection Assays

HIV-1_BaL_ was grown as described and was selected because R5 viruses predominate following sexual transmission [13], [14]. TZM-bl cells were cultured in 96 well dishes overnight as described [15]. The cells were infected with HIV-1_BaL_ (10^3^ TCID_50_) mixed 1∶1 with CVL or control buffer (normal saline containing 1.5 mg/mL bovine serum albumin). After 48 hour incubation at 37°C, the inoculum was removed by washing, cells were lysed and luciferase activity was measured in relative light units (RLU) [14]. Mock infected cells served as a negative control. All samples were tested in triplicate in two independent experiments.

To generate dose response curves to PRO 2000 under conditions that simulate the postcoital CVL, TZM-bl cells were infected with HIV-1_BaL_ diluted in media alone or in media containing pooled SP and mixed 1∶1 with serial dilutions of unformulated PRO 2000 diluted in pooled CVL. The pooled CVL and SP were generated from 3–5 healthy volunteers and processed as previously described [10]. Cell proliferation was assessed in uninfected plates treated with the CVL and pooled SP for 48 h using the CellTiter 96 cell-proliferation assay (Promega, Madison, WI) [14].

### Anti-HSV Activity of CVL

CaSki (human cervical epithelial) cells were infected with serial 10-fold dilutions of HSV-2(G) (0.01–1000 pfu/cell) mixed 1∶1 with each CVL or control buffer [16]. After 1-h incubation at 37°C, the cells were washed and overlaid with fresh medium. Plaques were counted after 48 hours by immunoassay [17]. Wells with plaques ranging from 20 to 150 were used to calculate the viral titer (PFU/ml). All samples were tested in duplicate in two independent experiments.

### Measurement of PRO 2000 Levels in CVL

The concentration of PRO 2000 in CVL supernatants was determined using a previously described fluorescence assay [16]. In brief, a standard stock was established by diluting 0.5% PRO 2000 gel into control CVL to a final concentration of 200 µg/ml and then serial 2-fold dilutions were generated for standards (range 0.1–100 µg/ml). 20 µl of each standard or test CVL samples were combined with 620 µg/ml of buffer (0.1 M sodium phosphate, pH 7.0, containing 0.1 M SDS) and fluorescence was measured (excitation 330 nm, emission 355 nm) in a fluorescence spectrophotometer (Perkin-Elmer model LS-5). The concentration of PRO 2000 in the samples was interpolated from the generated calibration curve.

### Statistical Analysis

GraphPad Prism (version 4; GraphPad Software) and SPSS for Windows (version 17) were used for statistical analyses. The antiviral activity in CVL was compared with Wilcoxon signed-rank tests to account for non-Gaussian distributions. Spearman's correlation coefficients were calculated to analyze correlations between PRO 2000 concentrations and mean antiviral activity in CVL. All tests for statistical significance were two sided, with p values≤0.05 considered significant.

## Results

### Subjects

Twenty heterosexual couples were assessed for eligibility. Ten couples were excluded; six had discordant HSV serologies, one female developed a genital herpes recurrence, one had an abnormal Pap test, and two had discordant HSV serologies and abnormal Pap tests. All enrolled participants completed the study as planned. Only three adverse events were reported, none of which was considered medically significant. One subject noted vaginal itching immediately following gel insertion; one with a prior history of intermenstrual bleeding noted vaginal spotting, and one developed rhinorrhea during the study. All participants were HSV-2 seronegative.

Demographics and clinical data for the enrolled couples are detailed in Table 1. The time between gel application and CVL collection for Visits 3 and 4 were similar and ranged between 40–105 minutes. Semen was detected in vaginal swabs obtained following intercourse (Visits 2 and 3) in all participants by a p30 immunoassay. The median (IQR) pH was significantly higher in the vaginal swab (p = 0.02) collected following sex (7.90 (6.90, 8.00)) compared to in the absence of sex (4.60 (4.50, 5.00)). PRO 2000 did not noticeably affect the vaginal pH (Table 1). In addition, the median (IQR) protein concentration (µg/ml) was significantly higher in postcoital CVL (Visits 2 and 3; 714 (339, 5091) and 720 (223, 6339)) compared to baseline CVL (296 (59, 492)) (p<0.01) (Table 1). Notably, the protein concentration was lower in the post-PRO 2000 (Visit 4; 39 (23, 188) CVL compared to baseline CVL (p = 0.014). The drug did not interfere with the assay as no differences in protein concentrations were observed if the standards were diluted in PBS or in PBS containing PRO 2000 gel (final concentration 100 µg/ml) (not shown). The concentrations may be lower in the post-gel CVL because the gel binds to mucosal proteins or cells, interfering with protein recovery by lavage.

**Table 1:** Demographic and clinical data from 10 couples.

Couple	Gender; Age; Race	HSV-1 Serostatus	Visit #	Time CVL (min)*	Vaginal pH before & after sex or gel	[Protein] in CVL (µg/ml)	[PRO 2000] in CVL (µg/ml)
1	F; 27; White	Neg	1			298	
	M; 32; White	Neg	2	40		297	
			3	65		340	3.7
			4	90		100	104.6
2	F; 31; Hawaiian	Neg	1			604	
	M; 31 Hawaiian	Neg	2	50		962	
			3	65		603	16.2
			4	65		174	28.25
3	F; 30; Asian	Pos	1			24	
	M; 32; Asian	Pos	2	35		95	
			3	90		105	3.7
			4	105		31.3	18.45
4	F; 29; Asian	Pos	1			505	
	M; 26; White	Pos	2	20	4; 8	494	
			3	60	4.5; 8	818	47.3
			4	65	4.5; 5	16	58.55
5	F; 25; White	Pos	1			478	
	M; 38; White	Pos	2	35	5; 8	5524	
			3	70	5; 8	3976	12.1
			4	70	5.5; 5	29	126.1
6	F; 23; Asian	Neg	1			93	
	M; 25; White	Neg	2	50	5; 8	6276	
			3	100	5; 8	5679	59.95
			4	100	5; 4.5	32	151.05
7	F; 30; White	Neg	1			0.5	
	M; 31; White	Neg	2	45	4.5; 7.5	736	
			3	65	4.5; 6.5	92	0.85
			4	60	4.5; 4	46	28.5
8	F; 26; White	Neg	1			112	
	M; 28; White	Neg	2	25	4.5; 8	4658	
			3	40	4.5; 7.8	6339	16.4
			4	40	4.5; 4.5	85	22.9
9	F; 25; Mixed	Neg	1			294	
	M; 24; White	Neg	2	60	5; 7	693	
			3	65	5; 6.8	1928	20.05
			4	65	4.9; 5.5	202	0
10	F; 25; White	Neg	1			378.5	
	M; 26; White	Neg	2	45	4.5; 6.4	379.8	
			3	55	4.7; 6.4	621.7	1.65
			4	65	4.6; 3.5	236.3	23.95

F, female; M, male; min, minutes.

* Time (minutes) between intercourse and CVL collection for Visit 2 and time after gel application of CVL collection for Visits 3 and 4.

### Anti-HIV Activity Is Lower in Post-Gel CVL Obtained following Coitus

**Figure 1:**
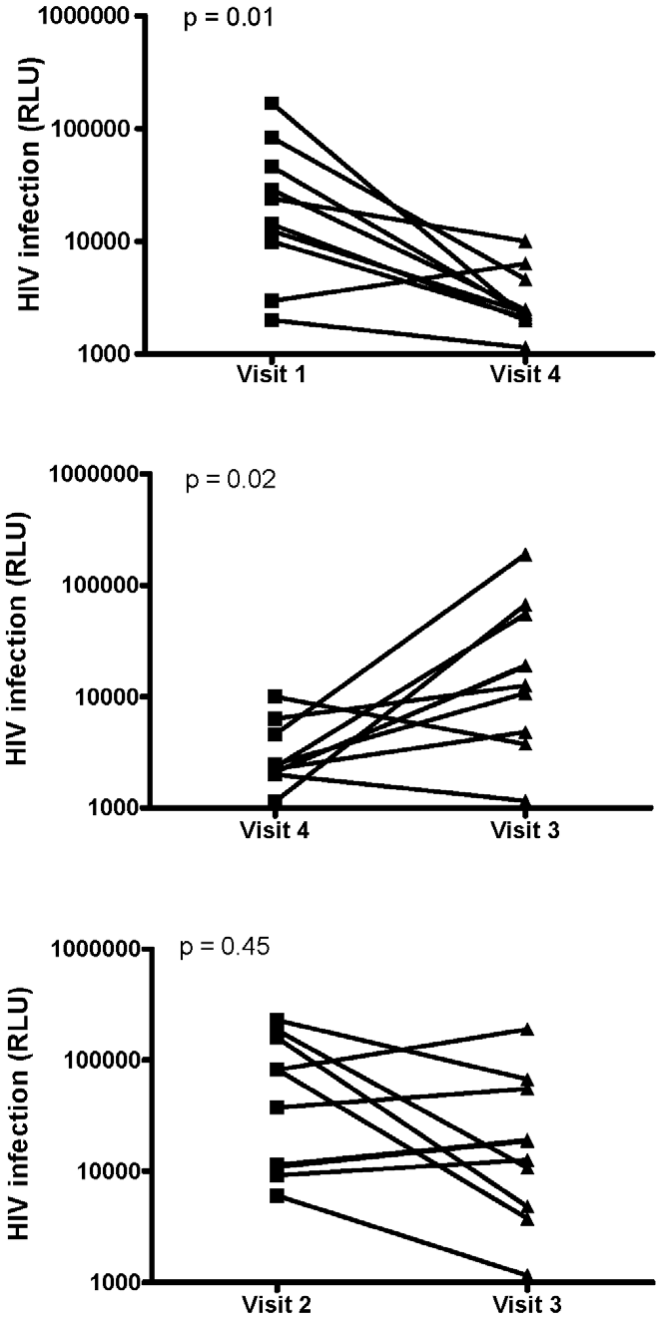
Loss in the anti-HIV activity in CVL collected following a single application of PRO 2000 gel in the absence or following coitus. TZM-bl indicator cells were exposed to 10^3^ TCID_50_ HIV-1_BaL_ in the presence of each CVL sample and after 48 h incubation, the cells were lysed and infection monitored by luciferase assay. Results are mean RLU obtained from 2 independent experiments, each conducted in triplicate. The upper panel shows results for each subject for Visit 1 (baseline) versus Visit 4 (post-gel), middle panel for Visit 4 (post-gel) versus Visit 3 (post-gel and postcoital) and lower panel for Visit 2 (postcoital) versus Visit 3 (post-gel and postcoital).

CVL collected following a single dose of PRO 2000 gel in the absence of coitus significantly inhibited HIV infection compared to baseline CVL (Visit 4 vs. Visit 1; p = 0.01) (Fig. 1, upper panel and Table 2). However, the inhibitory activity of post-gel CVL was significantly less following sex (Visit 4 vs. Visit 3; p = 0.02) (Fig. 1, middle panel) and there was no significant protection provided by post-gel, postcoital CVL (Visit 3) compared to postcoital CVL (Visit 2) (p = 0.45) (Fig. 1, lower panel). There was, however, substantial intercouple variation.

**Table 2:** Viral infection in presence of each CVL or control buffer.

Visit	HIV Infection RLU×10^3^ Median (IQR)*	HSV-2 Infection PFU/ml×10^3^ Median (IQR)*
Control buffer	172 (115, 222)	28,000 (13,625, 66,625)
Visit 1 (Baseline)	19.1 (8.2, 55.7)	10,150 (7138, 22,088)
Visit 2 (Postcoital)	60.0 (10.6, 165.8)	11,275 (6913, 15,950)
Visit 3 (Post-gel; postcoital)	15.7 (4.6, 58.3)	10,750 (1413, 34,475)
Visit 4 (Post-gel)	2.3 (2.1, 5.0)	8.35 (2.56, 58.38)

* IQR interquartile range.

**Figure 2:**
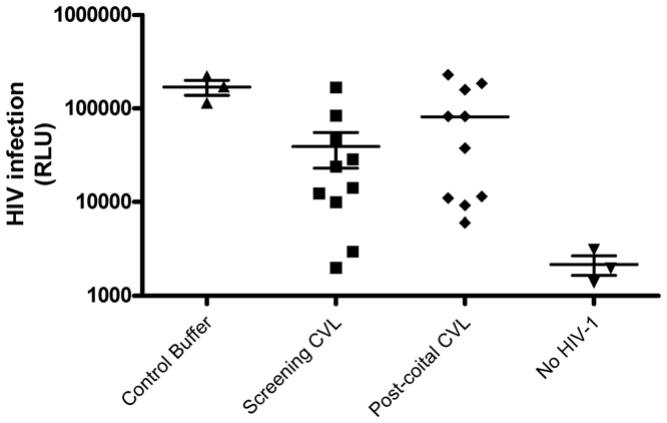
Endogenous anti-HIV activity in CVL collected in the absence or following coitus. TZM-bl cells were infected with 10^3^ TCID_50_ HIV-1_BaL_ in the presence of Visit 1 (baseline) or Visit 2 (postcoital) CVL or control buffer (saline with 1.5 mg/ml bovine serum albumin). Mock infected cells were included as controls. Results are mean RLU obtained from 2 independent experiments, each conducted in triplicate.

Notably, HIV infection was an order of magnitude lower (p = 0.2) (Fig. 2) when cells were infected in the presence of baseline CVL (Visit 1) compared to control buffer, which presumably reflects the endogenous activity of genital tract secretions. However, HIV infection was modestly higher (though not statistically significant, p = 0.17) in the presence of postcoital compared to baseline CVL (Visit 2 vs. Visit 1).

### Reduction in Anti-HSV Activity in Post-Gel CVL Obtained following Coitus

**Figure 3:**
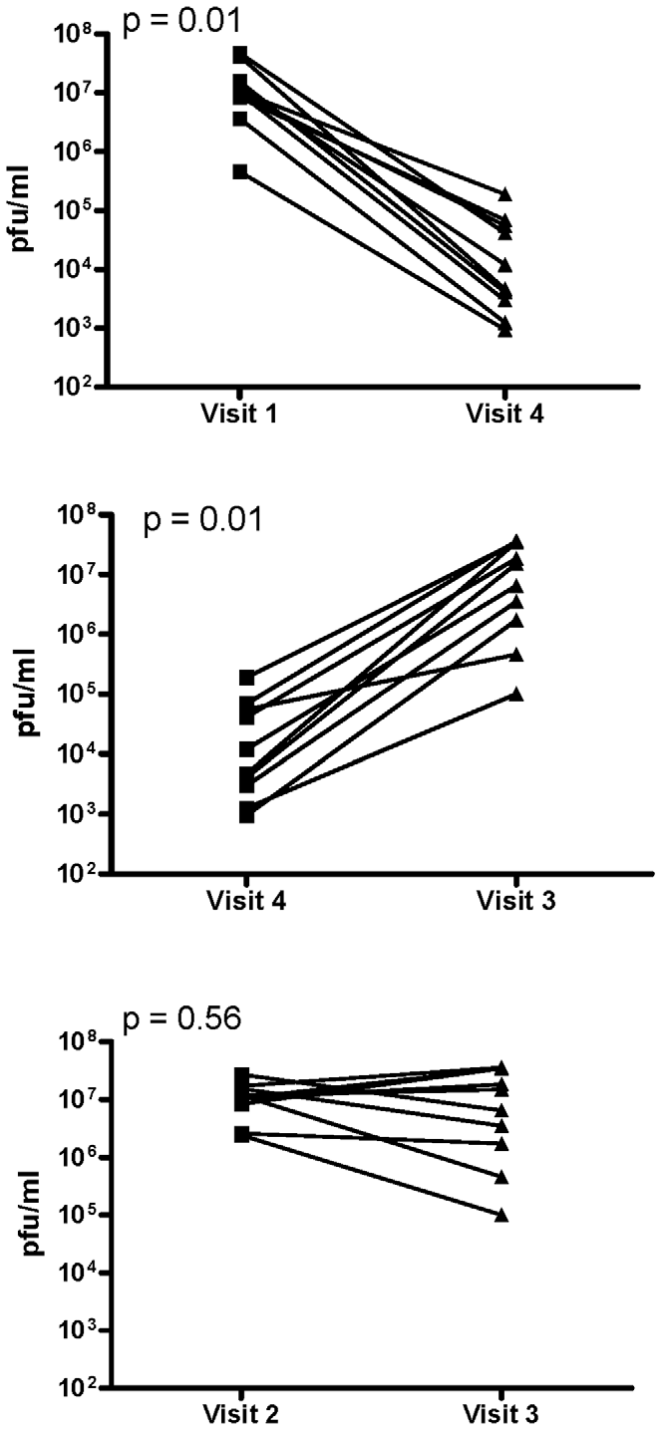
Loss in the anti-HSV activity in CVL collected following PRO 2000 gel application in the absence or following coitus. CaSki cells were infected with serial 10-fold dilutions of HSV-2(G) mixed with each CVL sample. After incubation for 2 h, the inoculum was removed by washing and the cells were overlaid with fresh media. Infection was monitored by counting plaques after 48 h. Results are viral titer (pfu/ml) calculated from 2 independent experiments where each sample was tested in duplicate. The upper panel shows results for each subject for Visit 1 (baseline) versus Visit 4 (post-gel), middle panel for Visit 4 (post-gel) versus Visit 3 (post-gel and postcoital) and lower panel for Visit 2 (postcoital) versus Visit 3 (post-gel and postcoital).

**Figure 4:**
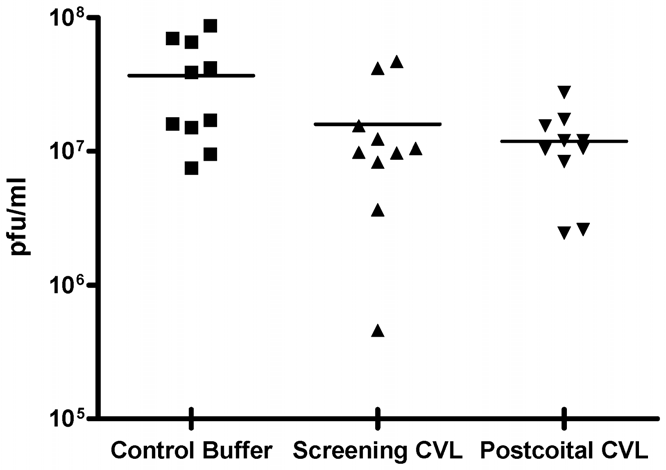
Endogenous anti-HSV activity in CVL collected in the absence or following coitus. CaSki cells were infected with HSV-2(G) in the presence of Visit 1 (baseline) or Visit 2 (postcoital) CVL or control buffer. Results are presented as viral titer (pfu/ml) obtained from 2 independent experiments, each conducted in duplicate.

Parallel studies were conducted with HSV-2. In the absence of coitus, the post-gel CVL (Visit 4) significantly inhibited infection (p = 0.01) and reduced viral titer ∼1000-fold compared to baseline CVL (Visit 1) (Fig. 3, upper panel and Table 2). However, there was significantly lower protective activity in CVL obtained following coitus (Visit 4 vs. Visit 3, Fig. 3, middle panel) (p = 0.01) and no significant difference in the antiviral activity of post-gel, postcoital CVL (Visit 3) compared to postcoital CVL (Visit 2) (p = 0.56; Fig. 3, lower panel). Notably, baseline CVL (Visit 1) significantly reduced HSV-2 plaque formation compared to control buffer and this endogenous activity persisted following coitus (p = 0.01; Fig. 4). None of the CVL samples had any impact on CaSki or TZM-bl cell viability (data not shown).

### PRO 2000 Concentrations

No significant differences were observed in the standard curves generated from serial dilutions of PRO 2000 gel mixed with pooled CVL or with a mixture of pooled CVL and SP (CVL and SP obtained from healthy donors, final concentration 20% SP), indicating that SP does not interfere with detection of PRO 2000. Notably, significantly lower drug was detected in CVL obtained following coitus (median (IQR) 14 (3, 27)) compared to CVL obtained in the absence of coitus (28 (22, 110) (p = 0.035; Table 1) and the antiviral activity correlated with the concentration of PRO 2000 (r = 0.48, p = 0.03 for both HIV and HSV-2). Thus, lower antiviral activity in postcoital CVL could be attributed, at least in part, to lower PRO 2000 concentrations. Whether the drug is redistributed within the genital tract, bound to semen, more difficult to recover because of physical changes to the gel or lost due to leakage cannot be differentiated. Only a few women reported leakage and these self-reports did not correlate with the concentration of PRO 2000 detected in CVL. In addition, variability in the volume of ejaculate may have impacted the amount of drug or genital tract secretions recovered in postcoital CVL.

**Figure 5:**
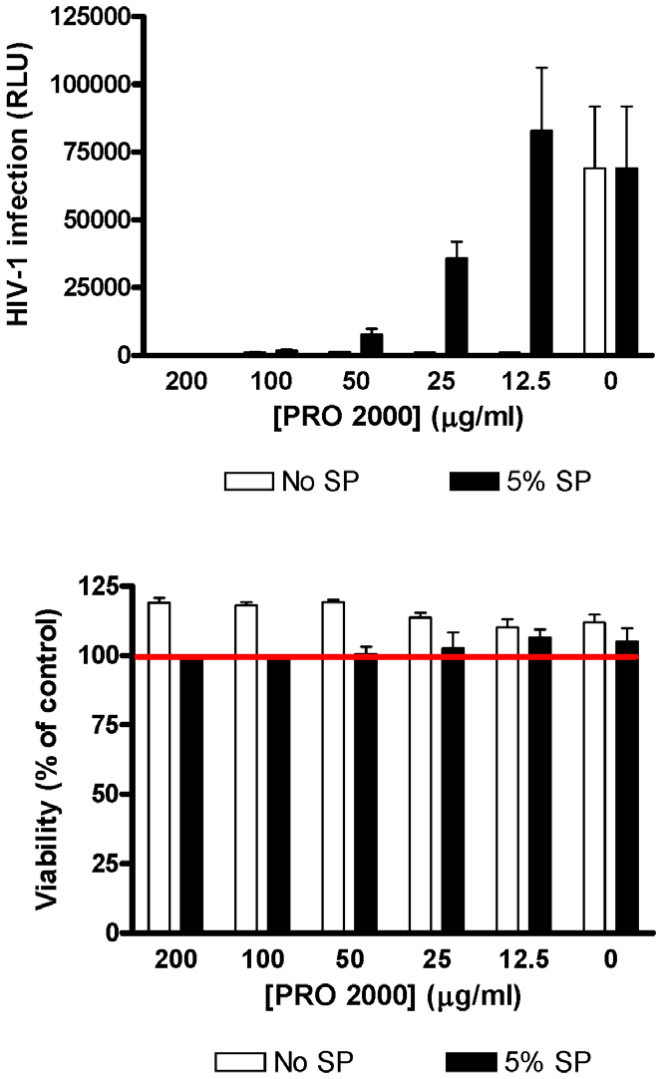
Seminal plasma interferes with anti-HIV activity of PRO 2000 in cell culture. TZM-bl cells were infected with HIV-1_BaL_ that had been diluted in media (no SP) or media containing 5% pooled SP and mixed 1∶1 with serial 2-fold dilutions of unformulated PRO 2000 diluted in pooled CVL obtained from healthy controls. Results are mean ± sd obtained from triplicate wells and are representative of 3 independent experiments (upper panel). Cell proliferation was assessed in parallel in uninfected plates treated with the CVL and pooled SP for 48 h (lower panel). Results are expressed as % viability (relative to mock-treated controls) and are means ± SD of 2 independent experiments where each condition was tested in triplicate.

However, lower drug concentrations do not fully explain the reduction in antiviral activity. For example, ∼50 µg/ml PRO 2000 was recovered in Visit 3 CVL from Couples 4 and 6, but little or no antiviral activity was detected, suggesting that SP interferes with the antiviral activity of PRO 2000 or enhances the infectiousness of virus. We previously published that PRO 2000 loses anti-HSV activity when virus is introduced in SP [10]. These *in vitro* studies were extended, focusing on anti-HIV activity. TZM-bl cells were incubated with serial 2-fold dilutions of PRO 2000 mixed in pooled CVL and infected with virus in media or in media containing 5% pooled SP. Higher concentrations of pooled SP (>12.5%) were cytotoxic when left in culture for 48 h (not shown). In the absence of SP, PRO 2000 completely inhibited HIV infection at a concentration less than 12.5 µg/ml, but >100 µg/ml was required to achieve complete protection when 5% SP was present (Fig. 5). The addition of 5% SP had little effect on HIV infectivity as the RLU were similar in the absence or presence of SP when no PRO 2000 was added.

## Discussion

This is the first study to evaluate the PK and PD of a candidate microbicide following coitus and clearly demonstrates that both differ significantly from results obtained in the absence of sexual intercourse. In addition to the significantly diminished antiviral activity (PD) and lower concentrations of PRO 2000 (PK), postcoital CVL had higher pH (mean 5.99 and 5.94 for visits 2 and 3, respectively) compared to CVL obtained in the absence of sex (pH 4.75 and 4.65 for visits 1 and 4, respectively). The postcoital CVL samples also had significantly greater protein concentrations compared to samples obtained in the absence of sex (Table 1). However, it is unlikely that these differences contributed to the differences in antiviral activity. No differences in the antiviral activity of PRO 2000 were observed across a broad pH range *in vitro* [18]. Moreover, a 1∶5 dilution of pooled seminal plasma (protein concentration ∼6 mg/ml) had little effect on viral infection [10].

The extent of antiviral activity and the optimal distribution of drug required for protection are unknown. The CVL may underestimate the total amount of drug found in the genital tract, some of which may have been lost in the cell pellet during centrifugation, adherent to epithelial cells within the genital tract, or inaccessible to lavage. In addition, drug distribution to non-target cells, could also result in drug dilution and decreased efficacy. Even at Visit 4, only a small percentage of administered PRO 2000 was recovered. However, the unbound drug may be most important. The primary mechanism by which PRO 2000 inhibits HIV infection is binding to viral gp120, although some activity may be attributed to binding to CD4 receptors on target immune cells [19]. If during sex, drug is redistributed, bound to cells in the ejaculate (which could include HIV-infected cells) or to target cells in the genital tract, it could provide some anti-HIV activity, although no protection was observed in the MDP301 trial. In contrast, redistribution of PRO 2000 might further diminish any impact against HSV-2, as infection likely occurs closer to the introitus (or outside the vagina) and the drug acts primarily by binding to the HSV-2 envelope glycoprotein B to competitively inhibit viral attachment and entry [18].

Not only did sexual intercourse result in a reduction in PRO 2000 recovered in CVL, but the *in vitro* data (Fig. 5 and [10]) suggest that SP interfere with antiviral activity. Even when the PRO 2000 concentrations in postcoital CVL (Visit 3) were similar to the concentration measured in non-postcoital samples (Visit 4) as observed with Couple 4, for example, the antiviral activity was lower.

Important limitations of this study are the single dose design, absence of a placebo gel group, reliance on CVL specimens alone, and small sample size. A single dose may underestimate the potential effectiveness of the drug, which may accumulate in the genital tract following repeated applications. In addition, differences in the duration of intercourse, ejaculate volume or composition may have contributed to variability in antiviral activity detected in postcoital samples. The addition of biopsy samples to CVL collection would provide information regarding drug distribution. Challenging biopsy samples *ex vivo* with HIV would be especially critical for evaluating microbicides that act intracellularly, such as tenofovir or dapivirine. However, the number, size, and site of sampling will require optimization as there is variability in the infectivity of biopsy tissue.

Although this study was not designed to evaluate endogenous antiviral activity in genital tract secretions, baseline CVL (Visit 1) inhibited both HIV and HSV-2, which is consistent with prior studies [20], [21], [22]. Notably, the anti-HIV activity was diminished when experiments were conducted with postcoital CVL (Visit 2), suggesting interference with the endogenous anti-HIV activity. Possibly, the alkaline pH or specific enzymes and proteases in semen inactivate, degrade, or interfere with protective immune mediators. In contrast, the anti-HSV-2 activity persisted in postcoital CVL (Visit 2). Differences in the viscosity between the control buffer and CVL samples may have contributed to the endogenous antiviral activity, although most of the mucous pellets with the cells during centrifugation. In addition, prior work indicates that the anti-HSV activity correlates with the concentration of specific antimicrobial proteins including defensins (human neutrophil peptides 1–3) and lysozyme [22], [23].

The endogenous anti-HSV activity in this study was less than previously observed [22] and may reflect the fact that all women who participated in this study were on hormonal contraception. In a recently completed study, we found that the anti-HSV-2 activity in CVL was reduced among women on hormonal contraception compared to women not using hormonal contraception [23]. Larger studies are needed to fully evaluate the genital tract environment both in the absence and following coitus.

In summary, this work demonstrates the feasibility and importance of conducting postcoital studies. The current paradigm of microbicide development should be modified to include postcoital sampling following single and repeated dosing with both active and placebo products and should be expanded to include both CVL and biopsies to more fully define the PK and PD of lead candidates prior to embarking on large-scale efficacy trials.
